# Simultaneous Analysis of Multiple *Mycobacterium tuberculosis* Knockdown Mutants *In Vitro* and *In Vivo*

**DOI:** 10.1371/journal.pone.0015667

**Published:** 2010-12-22

**Authors:** Antje Blumenthal, Carolina Trujillo, Sabine Ehrt, Dirk Schnappinger

**Affiliations:** Department of Microbiology and Immunology, Weill Cornell Medical College, New York, New York, United States of America; Unité de Génétique Mycobactérienne, Institut Pasteur, France

## Abstract

*Mycobacterium tuberculosis* (*Mtb*) represents one of the most persistent bacterial threats to human health and new drugs are needed to limit its impact. Conditional knockdown mutants can help validate new drug targets, but the analysis of individual mutants is laborious and time consuming. Here, we describe quantitative DNA tags (qTags) and their use to simultaneously analyze conditional *Mtb* knockdown mutants that allowed silencing the glyoxylate and methylcitrate cycles (via depletion of isocitrate lyase, ICL), the serine protease Rv3671c, and the core subunits of the mycobacterial proteasome, PrcB and PrcA. The impact of gene silencing in multi-strain cultures was determined by measuring the relative abundance of mutant-specific qTags with real-time PCR. This achieved accurate quantification over a broad range of qTag abundances and depletion of ICL, Rv3671c, or PrcBA resulted in the expected impairment of growth of *Mtb* with butyrate as the primary carbon source, survival during oxidative stress, acid stress and starvation. The impact of depleting ICL, Rv3671c, or PrcBA in multi-strain mouse infections was analyzed with two approaches. We first measured the relative abundance of mutant-specific qTags in total chromosomal DNA isolated from bacteria that were recovered from infected lungs on agar plates. We then developed a two-step amplification procedure, which allowed us to measure the abundances of individual mutants directly in infected lung tissue. Both strategies confirmed that inactivation of Rv3671c and PrcBA severely reduced persistence of *Mtb* in mice. The multi-strain infections furthermore suggested that silencing ICL not only prevented growth of *Mtb* during acute infections but also prevented survival of *Mtb* during chronic infections. Analyses of the ICL knockdown mutant in single-strain infections confirmed this and demonstrated that silencing of ICL during chronic infections impaired persistence of *Mtb* to the extent that the pathogen was cleared from the lungs of most mice.

## Introduction

The number of new tuberculosis (TB) cases is still rising and expected to reach a historical high of 9.8 million in 2010 [1]. This extraordinary impact on public health is in part due to drug-resistant *Mtb* strains, which have acquired resistance to four or more TB drugs. Such extensively drug-resistant (XDR) strains continue to emerge and spread, and are associated with particularly poor treatment outcomes [2]–[3]. Success rates for treating XDR-TB are generally between 30 and 50%, but mortality can reach 100% for outbreaks in patients co-infected with HIV [2], [4]. New drugs against TB are thus urgently needed.

The development of new TB drugs is hampered by the lack of knowledge about targets required for growth and survival of the pathogen *in vivo* and that are vulnerable to chemical inhibition. A variety of regulated expression systems, which can help overcome this obstacle, have recently been developed for mycobacteria [5]–[14]. Several of these system have been applied to characterize genes involved in processes as different as protein secretion [15]–[20], disulfide bond formation [21], DNA repair [22]–[23], cell division [5], [9], [24]–[26], proteolysis [27], cell envelope biosynthesis [28]–[30], DNA replication [31], carbon metabolism [32], and the stringent response [33]–[34]. A particularly useful application has been the construction of conditional knockdown mutants as it provides a genetic approach to study genes whose deletion prevents growth or causes death of the bacteria. Such conditional knockdown mutants helped assign functions to essential genes whose biological role was previously unknown. Examples for this include the identification of CarD as an essential regulator of rRNA transcription in mycobacteria [33] and the finding that the essential Esx-3 secretion system is required to acquire iron [16]–[17].

Controlled transcriptional silencing can also be used to identify genes that are required for growth and/or survival of *Mtb* during acute and chronic infections. For example, it was recently demonstrated that *Mtb* depends on *pckA*, which encodes the gluconeogenesis enzyme phosphoenolpyruvate carboxykinase (PEPCK), for growth during acute infections and also for survival during chronic mouse infections [32]. Genes like *pckA* that are required for survival of *Mtb* in many or all of the diverse conditions encountered during infections encode particularly attractive targets for the development of new TB drugs. Unfortunately, the high demands of animal experiments complicate the analysis of many conditional knockdown mutants in single-strain infections. We therefore set out to develop an approach that would allow us to quantitatively analyze several *Mtb* knockdown mutants simultaneously using multi-strain mixtures *in vitro* and in mouse infections.

## Results

### Construction of tagged conditional knockdown mutants for ICL, Rv3671c and PrcBA

**Figure 1:**
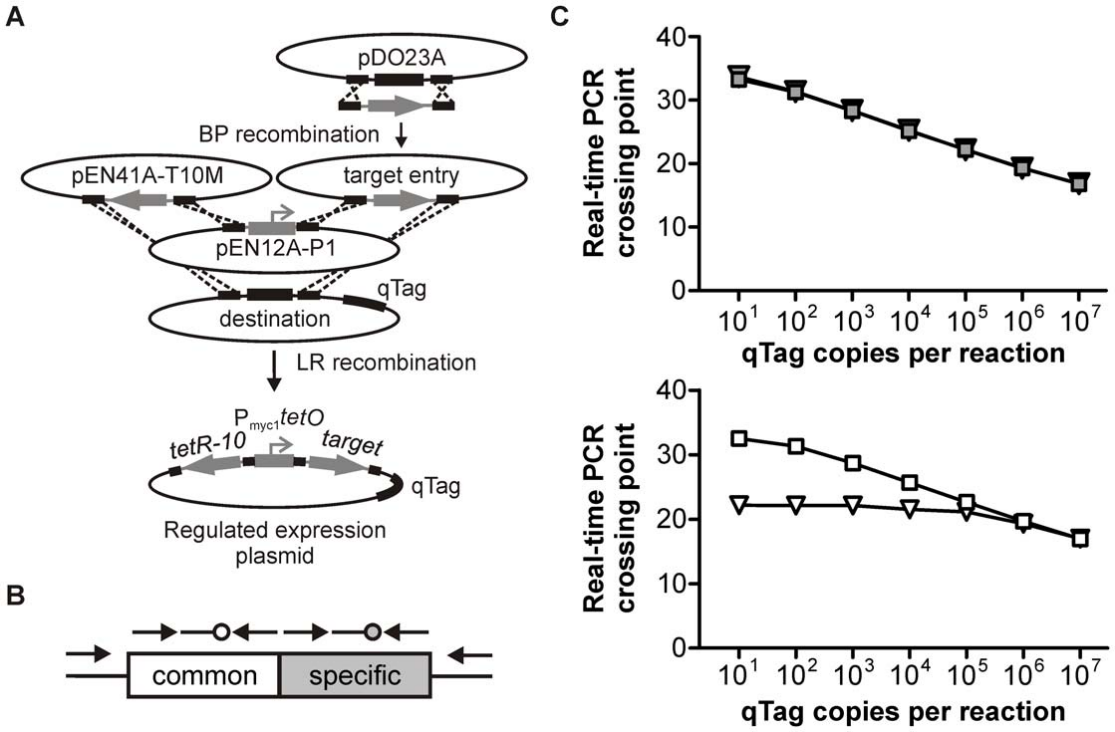
Cloning of mycobacterial expression plasmids by Gateway recombination, qTag design and qTag performance. (**A**) The gateway cloning system consists of *donor vectors*, which are used to clone PCR products by BP recombinations, *entry vectors*, which contain *tetR*, the TetR-controlled promoter P_myc1_ *tetO*, or the target gene whose transcription is to be regulated, and *destination vectors*, which are used to assemble (by LR recombinations) the three entry vectors into a regulated expression plasmid. (**B**) Each qTag contains targets for two different TaqMan primer/probe sets. The variable region is distinct in each tag and used to measure the relative abundance of different mutants. The common region is identical in each tag and used for normalization. The arrows located upstream and downstream the common and tag-specific regions represent the primers qTag-amp1 and qTag-amp2. (**C**) After transformation of *M. smegmatis* with an integrative plasmid containing qTag-17, chromosomal DNA was prepared and different concentrations were analyzed by real-time PCR using TaqMan probes that recognize the tag-specific region (upper panel) or the common region (lower panel). Real-time PCRs were performed in the presence (triangles) or absence (squares) of 10^5^ copies of qTag-24 and 10^5^ copies of qTag-26.

To facilitate the construction of conditional knockdown mutants we adapted MultiSite Gateway recombinational cloning [35] to the assembly of regulated expression plasmids for mycobacteria. The main components of the system we constructed are (i) a donor plasmid, pDO23A, which allows cloning target genes by BP recombinations [35], (ii) two entry plasmids, pEN41A-T10M and pEN12A-P1, which respectively contain *tetR-10* [6] and the TetR-controlled promoter P_myc1_ *tetO* [5], and (iii) several destination plasmids. To generate a regulated expression plasmid with this system, the target gene is first amplified using primers that contain attB2 and attB3 sites at their 5′ ends and recombined into pDO23A. After the DNA sequence of the resulting entry plasmid has been verified, *tetR-10*, P_myc1_ *tetO*, and the target gene are cloned into a destination plasmid by LR recombination (Figure 1.A). The destination plasmids described here all replicate episomally in *E. coli* and integrate into the phage L5 attachment site of mycobacterial chromosomes via site-specific recombination [36]. They differ in the antibiotic resistance that is used for their selection in mycobacteria (kanamycin resistance or streptomycin resistance) and the qTags that are located in the plasmid backbone downstream of the target gene. Each qTag consists of a constant region that is identical in all qTags and a variable region, which is tag-specific (Figure 1.A and Figure S1). The common and the variable region of each qTag are recognized by two TaqMan probes, which are labeled with different fluorophores and can be used together in the same real-time PCR. The qTags were designed so that the conserved amplicon and each of the tag-specific amplicons are 66 bp long and have a guanine/cytosine content between 53 and 59%. To analyze the performance of the qTags we integrated them into the *M. smegmatis* chromosome, prepared dilutions of the qTag-containing chromosomal DNA, and measured amplification of the constant and variable tags by real-time PCR. Figure 1.C displays data for one representative qTag and shows that the real-time PCR crossing points for amplification of the specific qTag region (Figure 1.C, gray squares) and the constant region (Figure 1.C, white squares) correlated well with the qTag copy numbers for reactions that contained between 10^1^ and 10^7^ qTag copies. The constant and specific regions of the five qTags used in this study all amplified with similar kinetics (data not shown). Reaction mixtures containing varying concentrations of one qTag and a constant amount of two other qTags demonstrated that amplification of the specific region was not affected by the presence of other tags (Figure 1.C, gray triangles). In contrast, PCRs with the TaqMan probe recognizing the constant region of each tag reflected the overall qTag concentration (Figure 1.C, white triangles).

We next determined if the tagged expression plasmids would allow us to analyze several conditional *Mtb* knockdown mutants simultaneously using multi-strain *in vitro* cultures. We selected isocitrate lyase (ICL), the serine protease Rv3671c, and the core subunits of the mycobacterial proteasome, PrcB and PrcA, as targets for mutant construction because silencing of these genes was expected to cause growth and/or survival defects in specific *in vitro* conditions and during mouse infections. ICL is required for the glyoxylate cycle, an anaplerotic reaction series that can replenish intermediates of the tricarboxylic acid (TCA) cycle when these are depleted by biosynthetic reactions, e.g. during growth on fatty acids [37]. *Mtb* ICL also has methylcitrate lyase activity, which is required for the methylcitrate cycle, a series of reactions that metabolizes propionyl-CoA into pyruvate [38]-[39]. Many *Mtb* strains contain two functional ICL genes, *icl1* and *icl2*. Deletion of *icl1* and *icl2* prevented growth of *Mtb* with fatty acids as the primary carbon source and also prevented growth of *Mtb* in mice [40]. Rv3671c is required for survival of *Mtb* during acid stress and for growth and persistence of *Mtb* in mice [41]. *PrcB* and *prcA* encode the beta and alpha subunits of the mycobacterial proteasome [42]. Depletion of PrcBA decreased resistance to nitrosative stress, increased resistance to oxidative stress and reduced persistence of *Mtb* in mice [43]. To construct conditional knockdown mutants for ICL and Rv3671c we cloned *icl1* and *rv3671c* as outlined in Figure 1 and integrated the expression vectors into the chromosomes of the knock out strains *Mtb* Δ*icl1* Δ*icl2* and *Mtb* Δ*rv3671c*, respectively. We constructed a tagged conditional knockdown mutant for PrcBA by integrating a qTag-containing *tetR* plasmid into the chromosome of H37Rv P_myc1_ *tetO*-*prcBA* in which the native *prcBA* upstream region had been replaced with P_myc1_ *tetO* [43]. TetR plasmids containing different qTags were also integrated into the chromosomes of *Mtb* H37Rv and *Mtb* Erdman to obtain wt-like reference strains. All conditional knockdown mutants were TetON mutants and expression of the targeted gene was induced with anhydrotetracycline (atc) or doxycycline (doxy) and repressed without atc/doxy.

### Growth of *rv3671c*-TetON, *prcBA*-TetON, and *icl*-TetON with different carbon sources

**Figure 2:**
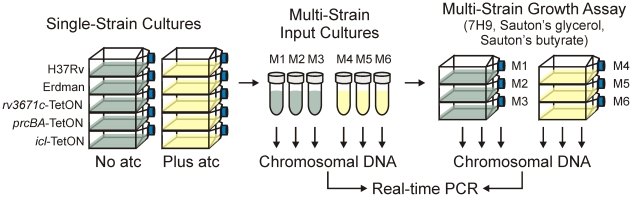
Experimental design to measure growth of multiple mutants in multi-strain liquid cultures. M1 to M6 stands for mix 1 to mix 6. A detailed description of the experimental design is in the main text.

The experimental design we used to measure the impact of silencing *rv3671c*, *prcBA*, or *icl1* on growth of *Mtb* in multi-strain liquid cultures is illustrated in Figure 2. Each of the three knockdown mutants and the two reference strains were grown separately before equal amounts of each strain were mixed and used to inoculate (i) 7H9 medium, (ii) Sauton's medium with glycerol as the primary carbon source, and (iii) Sauton's medium with butyrate as the primary carbon source. For each medium, chromosomal DNA was prepared from triplicate cultures after different periods of incubation at 37°C. The relative abundance of a specific qTag (which corresponds to the relative abundance of a specific mutant in the strain mix) was calculated based on real-time PCRs that each contained two TaqMan probes: one to detect the region that is identical in each tag and one to detect the tag-specific region. This combination of two TaqMan probes allowed us to measure, in the same reaction, the total amount of chromosomal DNA and the fraction of chromosomal DNA that stemmed from an individual mutant.

**Figure 3:**
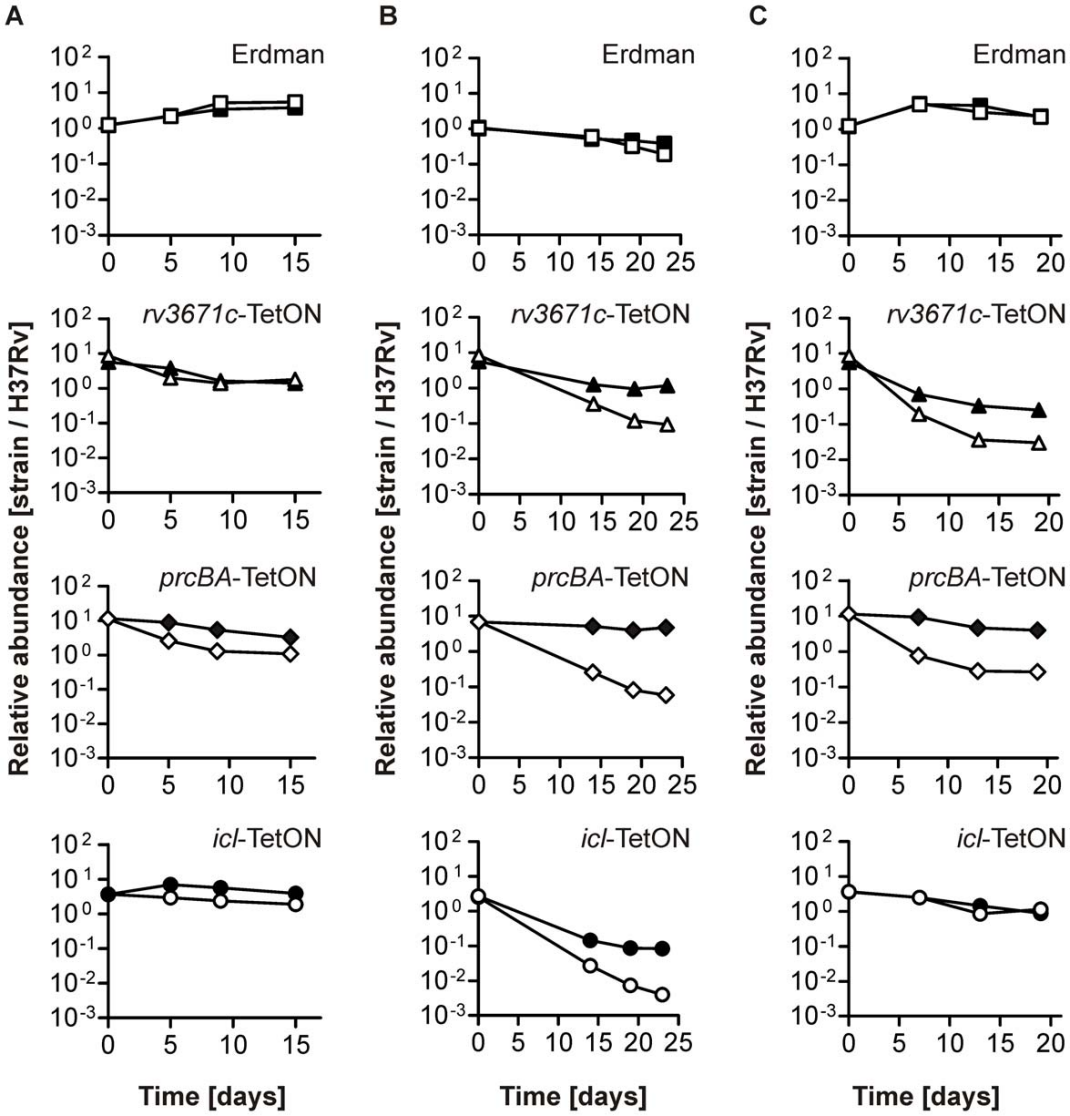
Growth of H37Rv, Erdman, *rv3671c*-TetON, *prcBA*-TetON, and *icl*-TetON in multi-strain liquid cultures. Multi-strain cultures were prepared, grown in 7H9 medium (**A**), Sauton's medium containing butyrate (**B**) or glycerol (**C**) and analyzed as indicated in Figure 2. Black and clear symbols identify data generated from atc-containing or atc-free cultures, respectively. Data are averages of triplicate cultures and representative of at least two independent experiments. Error bars (representing standard deviations) cannot be seen because they are smaller than the data point symbols.

**Figure 4:**
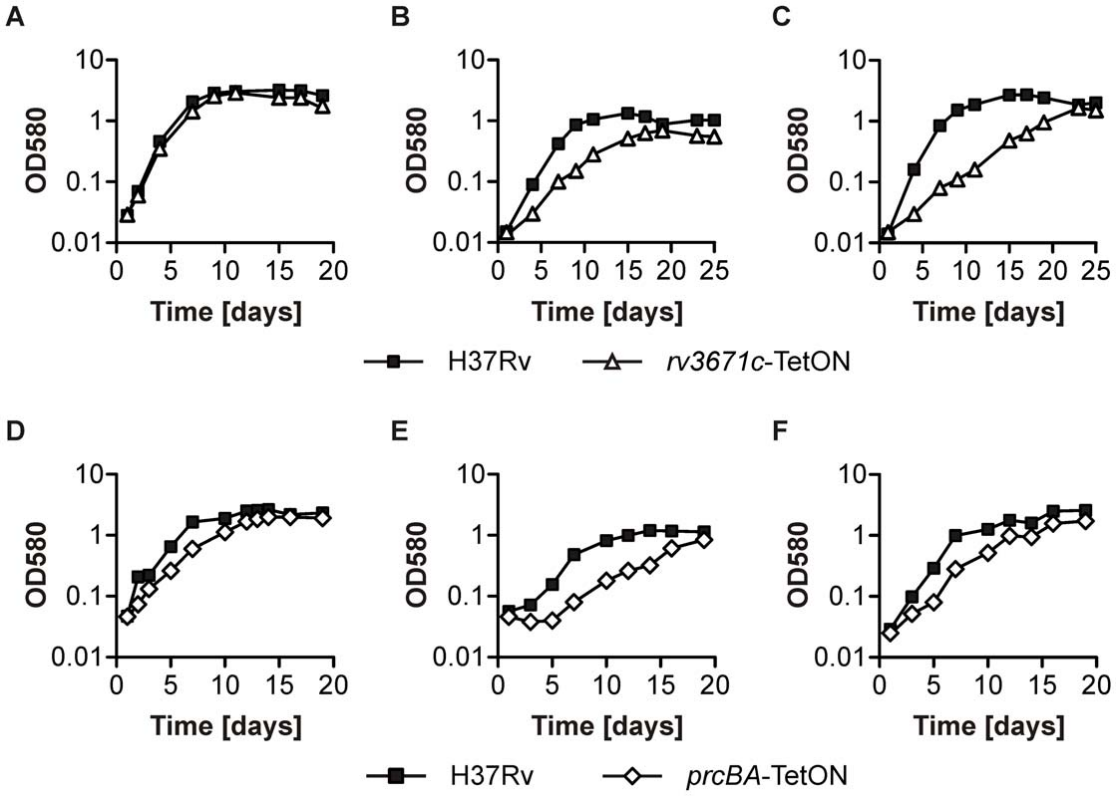
Impact of silencing *rv3671c* or *prcBA* on growth of *Mtb* in different liquid media. Growth of *Mtb* H37Rv (squares), *rv3671c*-TetON (triangles), *prcBA*-TetON (diamonds) without atc was analyzed in 7H9 medium (**A,D**), Sauton's medium containing butyrate (**B,E**) or glycerol (**C,F**) as the primary carbon source using optical density measurements at the indicated time points. Data represent results of three (*rv3671c*-TetON) and two (*prcBA*-TetON) independent experiments.

Figure 3 shows the relative abundances of *Mtb* Erdman, *icl*-TetON, *prcBA*-TetON and *rv3671c*-TetON normalized to that of H37Rv. White and black symbols represent data from atc-free and atc-containing cultures, respectively. As expected, we found (i) that atc did not alter the relative growth rates of Erdman, of *icl*-TetON in medium containing glycerol as the primary carbon source [40], and of *rv3671c*-TetON in 7H9 medium [41], (ii) that growth of *prcBA*-TetON in 7H9 was slightly reduced without atc [43], and (iii) that growth of *icl*-TetON without atc was reduced with butyrate as the primary carbon source [40]. *Icl*-TetON grew better with atc in Sauton's-butyrate than without atc but it did not reach the growth rate of H37Rv. How inactivation of *rv3671c* or *prcBA* would impact growth of *Mtb* in Sauton's medium had not been determined previously. The multi-strain experiments suggested that silencing of *rv3671c* caused a growth defect and that the slight growth impairment of *prcBA*-TetON in 7H9 medium was amplified in Sauton's medium. Both of these predictions were verified in experiments in which we used optical density measurements to follow growth of single-strain cultures (Figure 4).

### Survival of *rv3671c*-TetON, *prcBA*-TetON, and *icl*-TetON during acid stress, oxidative stress, and starvation

**Figure 5:**
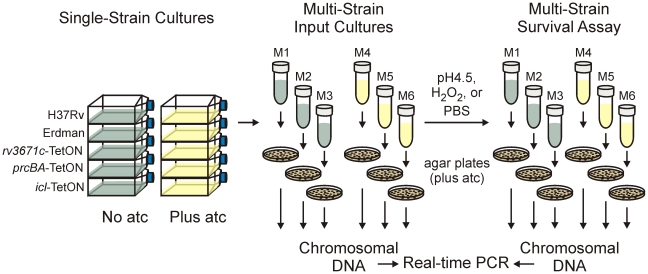
Experimental design to analyze survival of multiple mutants in multi-strain cultures. M1 to M6 stands for mix 1 to mix 6. A detailed description of the experimental design is in the main text.

**Figure 6:**
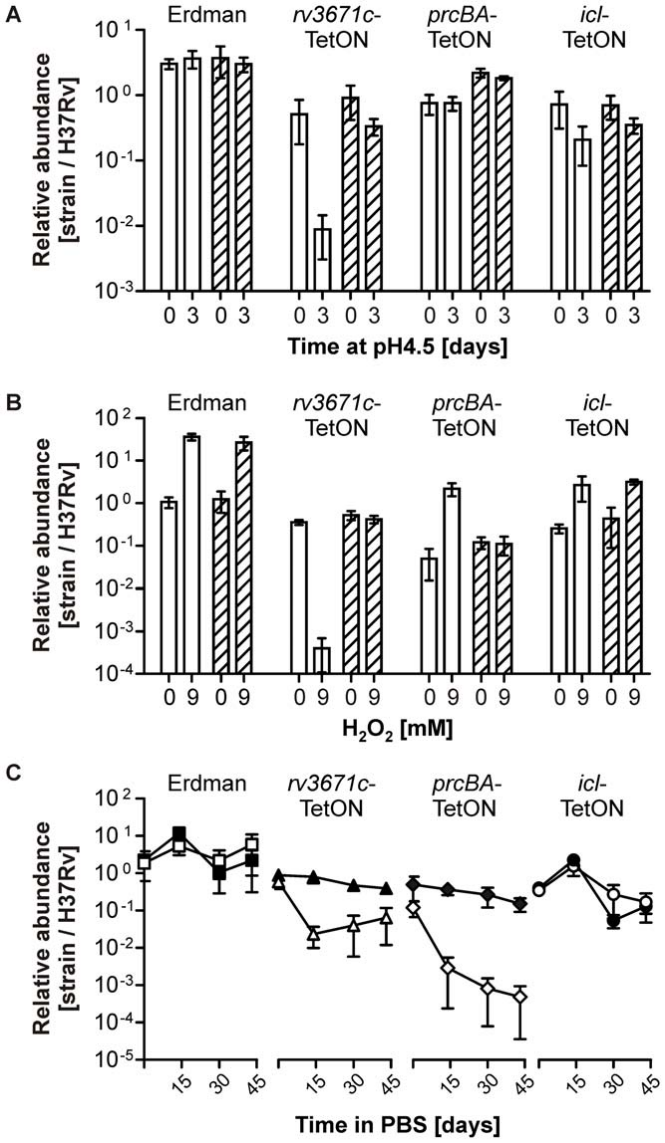
Survival of H37Rv, Erdman, *rv3671c*-TetON, *prcBA*-TetON, and *icl*-TetON in multi-strain liquid cultures. Multi-strain cultures were prepared and exposed to different stresses as described in Figure 5. (**A**) The relative abundance of each strain was measured before and after three days of acid stress (pH 4.5) in the absence (clear bars) or presence (hatched bars) of atc. (**B**) Cultures were treated with 9 mM H_2_O_2_ for four hours and compared to untreated controls. (**C**) Cultures were starved for the indicated times in PBS with (black symbols) or without (clear symbols) atc. Data in (A), (B) and (C) are averages of at least four cultures and representative of at least two independent experiments. Error bars represent standard deviations.

**Figure 7:**
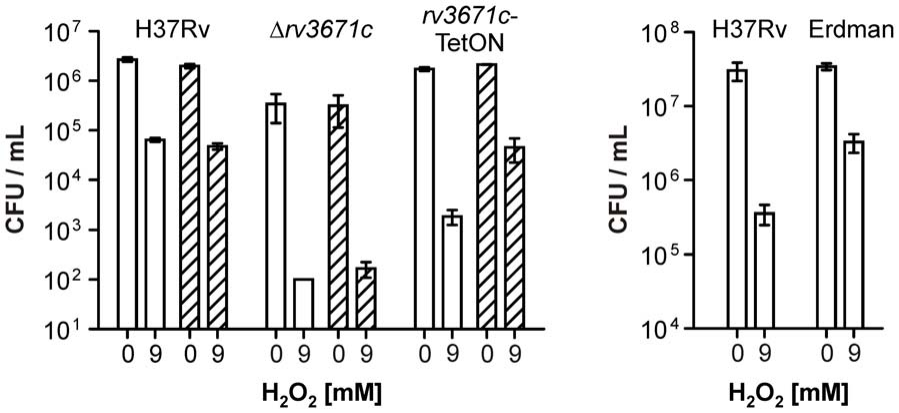
Survival of H37Rv, Δ*rv3671c*, *rv3671c*-TetON, and Erdman during oxidative stress. CFU recovered from single-strain cultures grown without (clear bars) or with (hatched bars) atc after exposure to 9 mM H_2_O_2_ for four hours were compared to CFU recovered from untreated controls. Data are averages of triplicate cultures and representative of at least two independent experiments. Error bars represent standard deviations.

Deletion of *rv3671c* impaired resistance of *Mtb* to acid [41] and inactivation of *prcBA* decreased survival during starvation in PBS [27] and increased resistance to oxidative stress [43]. To determine if we could recapitulate these survival defects in multi-strain cultures we used an experimental design in which chromosomal DNA was prepared not directly from liquid cultures but instead from bacteria that survived the stress treatment and were recovered on agar plates (Figure 5). In accordance with the phenotypes that have been reported for the corresponding deletion and transposon mutants [27], [41], silencing of *rv3671c* decreased survival of *Mtb* during acid stress and silencing of *prcBA* decreased survival during starvation and increased survival during oxidative stress (Figure 6). All of these phenotypes were complemented by atc. The multi-strain experiments also suggested that silencing of *rv3671c* decreased resistance of *Mtb* against H_2_O_2_ and that the *Mtb* Erdman reference strain was more resistant to H_2_O_2_ than the *Mtb* H37Rv reference strain. These findings were confirmed in single-strain experiments (Figure 7).

### Growth and survival of *rv3671c*-TetON, *prcBA*-TetON, and *icl*-TetON during multi-strain mouse infections

**Figure 8:**
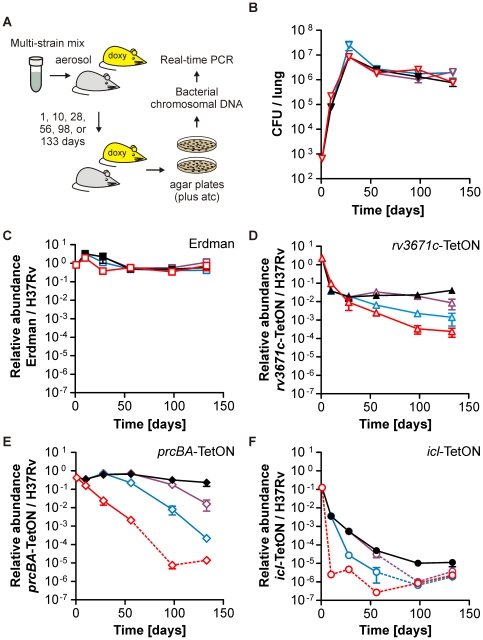
Growth and survival of H37Rv, Erdman, *rv3671c*-TetON, *prcBA*-TetON, and *icl*-TetON with and without doxy in mice. Mice were infected with the five-strain mix by aerosol and chromosomal DNA was prepared form bacteria recovered from lungs on agar plates. Lung extracts were plated 1, 10, 28, 56, 98 and 133 days post infection. Mice either received doxy throughout the infection (black symbols), from day 1 to day 10 (blue symbols), from day 1 to day 28 (purple symbols) or not at all (red symbols). Data are averages from four to five mice per group; error bars represent the standard error of the mean. Dotted lines end in data points for which the relative abundance of a specific mutant was below the limit of detection in the majority of the mice. (**A**) Experimental design. (**B**) Total CFU per lung. Relative abundance of Erdman (**C**), *rv3671c*-TetON (**D**), *prcBA*-TetON (**E**), and *icl*-TetON (**F**).

We combined equal amounts of *rv3671c*-TetON, *prcBA*-TetON, *icl*-TetON, and the reference strains and infected mice with aerosols containing all five strains. Mice were sacrificed 1, 10, 28, 56, 98 and 133 days post infection and lung homogenates were spread on atc-containing agar plates to enumerate colony forming units (CFUs) and to prepare chromosomal DNA (Figure 8.A). Doxy was administered either throughout the infection, from days 1 to 10, from days 1 to 28, or not at all. Doxy had no impact on the total CFUs isolated from mouse lungs (Figure 8.B). Chromosomal DNA was prepared from bacteria that were recovered from the agar plates after incubation at 37°C for three to four weeks and used to measure the abundance of each strain relative to *Mtb* H37Rv. The relative abundance of *Mtb* Erdman 10 days post infection was approximately 5-fold higher than one day post infection, varied at 28 days post infection, and after 56, 98 and 133 days of infection corresponded to that of day one irrespectively of the presence or absence of doxy (Figure 8.C). The two reference strains thus behaved similarly in mice and their relative abundances were not affected by doxy. An *Mtb rv3671c* transposon mutant was strongly attenuated in mice [41]. Without doxy, the relative abundance of *rv3671c*-TetON had decreased to less than 0.001% of that of H37Rv at the end of the multi-strain infection (Figure 8.D). Removal of doxy after 10 or 28 days caused less severe attenuation. These defects were partially complemented with doxy. Silencing of *prcBA* in single-strain mouse infections strongly attenuated survival of *Mtb* during chronic infections but had less impact during growth of *Mtb* during acute infections [43]. In the multi-strain infection described here, the relative abundance of *prcBA*-TetON in mice that did not receive doxy was also close to that of the H37Rv reference strain at day 10 post infections, but decreased drastically at later time points and was at or below the limit of detection 98 and 133 days post infection (Figure 8.E). *PrcBA*-TetON specific PCR signals decreased with similar kinetics irrespectively of the time at which silencing of *prcBA* was initiated. The attenuation of *prcBA*-TetON was fully complemented with doxy. Depletion of ICL had the most drastic impact on growth and survival of *Mtb* (Figure 8.F). The relative abundance of *icl*-TetON was at or below the limit of detection already after 10 days in mice that did not receive doxy. As we had observed in Sauton's medium with butyrate, the growth defect of *icl*-TetON in mice was not fully complemented with doxy. Removal of doxy after 10 or 28 days also left the relative abundance for *icl*-TetON below the limit of detection in most mice at later time points. Taken together, the results from the multi-strain infection were in good agreement with those of the previously reported single-strain infections. However, silencing of *icl1* in *icl*-TetON had a much more drastic impact on persistence of *Mtb* in mice than was caused by the deletion of *icl1* alone [44].

### Measuring the relative abundance of individual mutants directly from infected lung tissue

**Figure 9:**
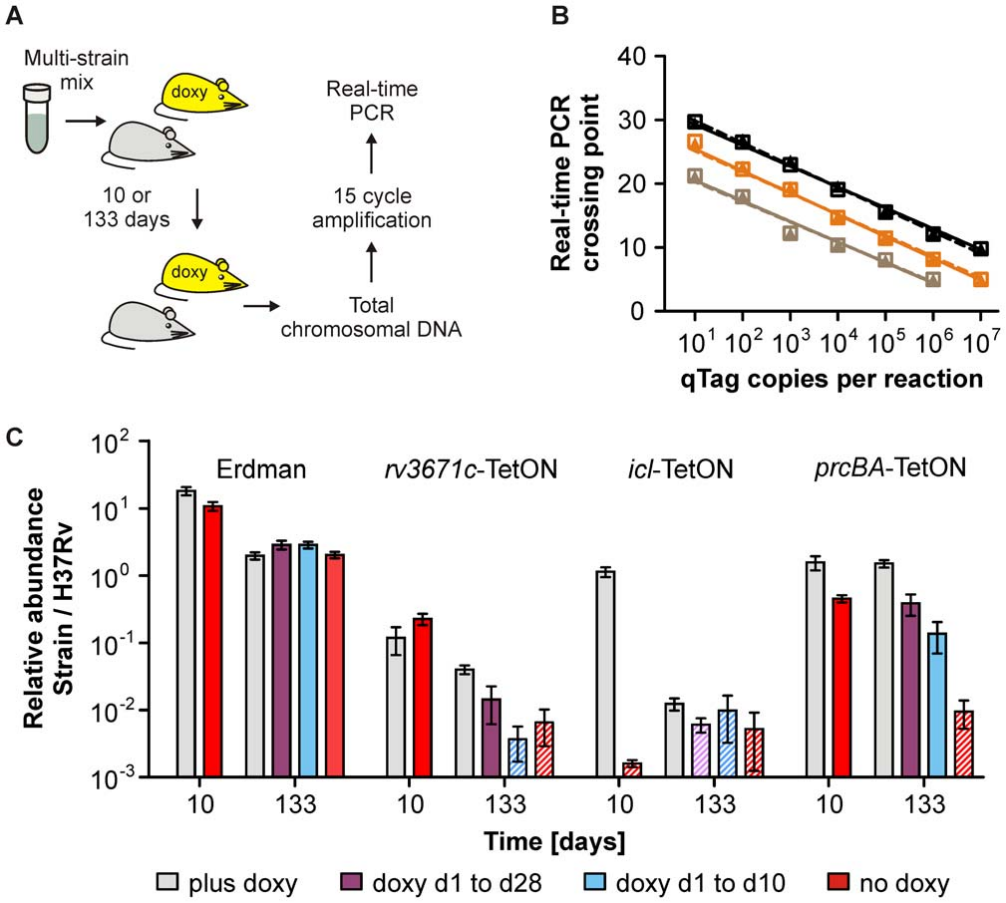
Quantification of strain abundances directly from infected lungs. (**A**) Lungs of mice that had been infected for 10 or 133 days were used to prepare total chromosomal DNA, which contained bacterial DNA as well as host DNA. This DNA was analyzed after amplification with primers qTag-amp1 and qTag-amp2. (**B**) The indicated copy numbers of one purified, qTag-containing plasmid were analyzed by real-time PCR after the qTags had been amplified with qTag-amp1 and qTag-amp2 for 10 (black), 15 (orange) or 20 PCR cycles (brown). Triangles and dotted lines represent measurements of the constant tag region; squares and solid lines represent quantifications of the variable tag region. (**C**) Relative abundance of *Mtb* Erdman, *rv3671c*-TetON, *icl*-TetON, and *prcBA*-TetON as measured from total lung extracts after amplification with qTag-amp1 and qTag-amp2 for 15 PCR cycles. Data are averages from four to five mice; error bars represent the standard deviations. Hatched bars indicate data points for which the relative abundance of a mutant was at or below the limit of detection in at least half of the mice.

The procedure we had used to analyze the mutants in mice required us to cultivate the bacteria that were isolated from lungs on agar plates for three to four weeks prior to the isolation of chromosomal DNA. To determine if we could measure the relative abundance of individual mutants without first growing them on agar plates, we also prepared total DNA directly from infected lungs (Figure 9.A). Real-time PCR analysis of these samples indicated that the fraction of *Mtb* DNA was too low to reproducibly quantify the abundances of different mutants (data not shown). We therefore tested if a two-step PCR method developed to quantify low-abundance transcripts from biopsy samples [45] could be adapted to increase the sensitivity of our analysis. We designed the primers qTag-amp1 and qTag-amp2 (Figure 1B), which hybridize to conserved regions upstream and downstream of each qTag, and analyzed first if PCRs with these primers could reproducibly amplify the different qTags. Reactions containing different amounts of purified qTag-containing plasmids showed that 10, 15 and 20 cycles of PCR increased the amount of the constant and the tag-specific qTag according to the number of PCR cycles and irrespectively of the initial concentration of the qTag (Figure 9.B). Next, we used DNA prepared from lungs as templates in a 15-cycle PCR with qTag-amp1 and qTag-amp2 to simultaneously amplify all qTags. Subsequent measurements of the relative abundance of each qTag after this pre-amplification were performed by real-time PCR. DNA from mouse lungs 10 days post infection indicated (i) an increased abundance of *Mtb* Erdman compared to *Mtb* H37Rv, (ii) a decreased abundance of *rv3671c*-TetON, (iii) a drastic decrease of *icl*-TetON without doxy, and (iv) a slight attenuation of *prcBA*-TetON without doxy (Figure 9.C). The relative abundance on day 10 post infection of *icl*-TetON in mice that received doxy was higher than what we had observed when the samples were analyzed after outgrowth on agar plates. This might be due to stability of the chromosomal DNA from dead bacteria within mouse lungs [44]. However, there was no significant difference in the relative abundance of *rv3671c-*TetON or *prcBA*-TetON when measured directly from the lungs or after outgrowth on plates. Thus death of some mutants might be accompanied by faster degradation of chromosomal DNA than death of others.

In mouse lungs prepared 133 days post infection the abundance of *Mtb* Erdman was very similar to that of *Mtb* H37Rv; *rv3671c*-TetON and *icl*-TetON were strongly attenuated, and the abundance of *prcBA*-TetON decreased as the period of time during which the bacteria were in mice without doxy increased. Overall, these findings are in good agreement with those from chromosomal DNAs prepared from bacteria that were recovered from lungs on agar plates. The main difference was in the limit of detection; while chromosomal DNA from bacteria that were grown on agar plates allowed to measure mutants whose abundance was at least 0.0001% of H37Rv, between 0.01% and 0.001% had to be present to allow detection in the DNA that was directly isolated from lungs.

### Inactivation of isocitrate lyase activity severely attenuates *Mtb* during acute and chronic mouse infections

**Figure 10:**
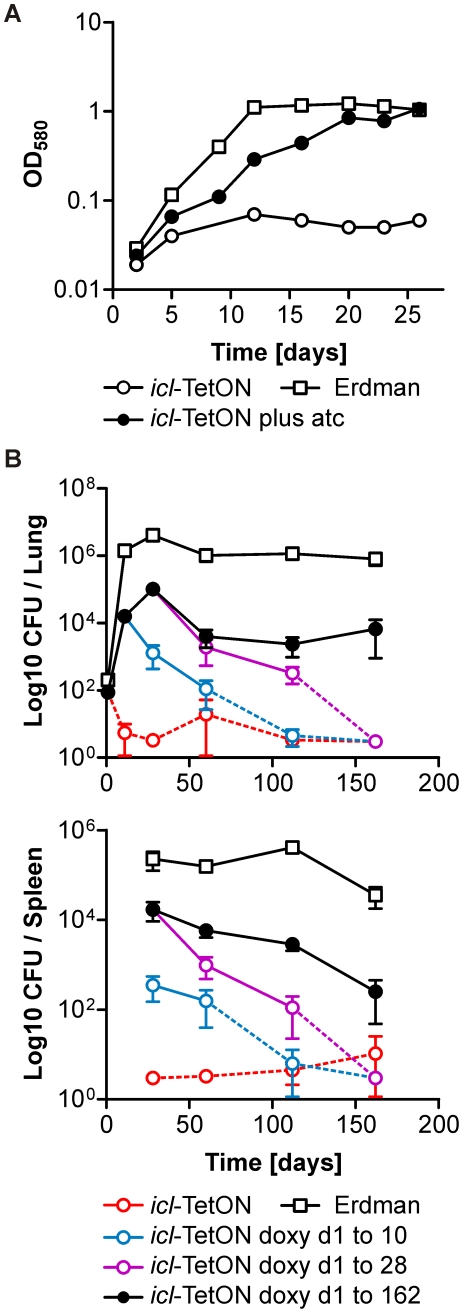
*Mtb icl*-TetON. (**A**) Growth with butyrate as the primary carbon source. *Mtb icl*-TetON (circles) was cultivated with atc (black circles) or without atc (white circles) in modified Sauton's medium containing butyrate as the primary carbon source. Growth of *Mtb* Erdman was analyzed without atc (white squares). Data are represenative of three independent experiments. (**B**) Impact of silencing *icl* on growth and persistence of *Mtb* in mouse lungs. *Mtb* Erdman (white squares) was analyzed in mice that did not receive doxy. *Mtb icl*-TetON was analyzed in mice that did not receive doxy (red circles), received doxy from day 1 to day 10 (blue circles), from day 1 to day 28 (purple circles), or received doxy throughout the infection (black circles). Data are averages from four mice; error bars represent the standard error of the mean. The limit of detection was 4 CFUs per lung. Dotted lines end in data points for which most of the lungs contained 4 or fewer CFUs. (**C**) Impact of silencing *icl* on persistence of *Mtb* in mouse spleens. As described for (B).

Deletion of *icl1* and *icl2* prevented growth of *Mtb* in mice [40]. *Mtb* Δ*icl1* Δ*icl2* could thus not be used to determine how inactivation of both isocitrate lyases would affect persistence of *Mtb* during established, chronic infections. Deletion of only *icl1* impaired persistence of *Mtb* in mice such that the CFUs isolated from lungs during chronic infections were reduced by approximately 90% [40], [44]. In contrast, silencing of *icl1* in *icl*-TetON reduced the relative abundance of this mutant by more than 99.999% compared to the reference strains in our multi-strain infection, even if silencing was initiated 28 days post infection. *Icl2* was deleted in our *icl*-TetON strain but had remained intact in *Mtb* Δ*icl1* [44]. This suggested that ICL2 might have masked the impact the complete inactivation of ICL activity has on persistence of *Mtb* during chronic mouse infections. Alternatively, the more severe phenotype of *icl*-TetON could have resulted from a competition with other strains in the multi-strain infection. To distinguish between these possibilities we performed single-strain experiments with *icl*-TetON. First we measured growth of *icl*-TetON in Sauton's medium with butyrate using optical density measurements. As expected, *Mtb icl*-TetON did not grow in medium that contained a fatty acid (butyrate) as the primary carbon source without atc (Figure 10.A). This growth defect was complemented with atc but growth of *Mtb icl*-TetON remained slower than that of the reference strain in which *icl1* and *icl2* were intact. Growth of *Mtb icl*-TetON in medium containing glucose and/or glycerol as the primary carbon source was similar to growth of the reference strain and growth of the reference strain was not affected by atc in any of the media (data not shown).

Next, we infected mice with *Mtb icl*-TetON and determined CFUs isolated from lungs and spleens at different times post infection. Doxy was used to induce ICL1 expression during these infections. In mice that did not receive doxy, the course of the *Mtb icl*-TetON infection was very similar to that reported for *Mtb* Δ*icl1* Δ*icl2* [40] (Figure 10.B); only lungs from mice sacrificed one day post infection yielded CFUs that were significantly above the limit of detection of four CFUs per lung. On average, less than four CFUs were isolated from no-doxy mice 10, 28, 112 and 162 days post infection. Similarly to what we observed *in vitro* and in the multi-strain infections, addition of inducer did not restore growth to that of the reference strain. However, with doxy *Mtb icl*-TetON grew from ∼10^2^ CFUs on day one to ∼10^5^ CFUs on day 21, and CFUs remained at ∼10^4^ per lung on day 56 and thereafter. In contrast, removing doxy from the diet of the infected mice on day 10 or day 28 post infections caused a steady decline in the CFUs and cleared the infections in most mice (Figure 10.B). Repression of ICL had a similar impact on the survival of *Mtb icl*-TetON in spleens (Figure 10.C). These data are in agreement with the multi-strain infection and indicate that inactivation of *icl1* and *icl2* severely attenuated *Mtb* during both the acute and chronic phase of infection.

## Discussion

The experimental strategy described here resembles transposon site hybridization (TRASH) [46]-[48] and signature tagged mutagenesis (STM) [49] in so far as it also allows analyzing different mutants simultaneously. However, in contrast to TRASH and STM it is not tied to a specific type of mutagenesis. TRASH and STM, which have been applied to study *Mtb* gene functions with great success [47]–[48], [50]–[55], use transposons for both, mutagenesis and mutant labeling. The ease with which transposon mutants can be generated allows applying TRASH and STM to bacterial populations containing dozens to thousands of different mutants. Such complex mutant pools can, however, not be analyzed in some experimental settings. For example, aerosol infections resembling natural transmission cannot be performed with highly diverse bacterial populations. In addition, the hybridization based methods that are generally used to measure the abundance of individual mutants by STM or TRASH, require relative large amounts of DNA or RNA and exhibit a limited dynamic range. Our goal was to develop an approach for measuring the relative abundance of individual mutants in mutant mixtures that can be applied to any kind of mutant, is highly sensitive, has a large dynamic range and allows to accurately measure growth and survival of *Mtb* mutants in mouse lungs after aerosol infection.

The qTags we developed for this purpose have several features which make them suitable to analyze multi-strain cultures: First, their abundance can be accurately measured by real-time PCR over a concentration range spanning at least six orders of magnitude (Figure 1.C). Second, they contain a conserved region and a tag-specific region (Figure 1.B), which include binding sites for two TaqMan probes that are labeled with different fluorophores and can thus be analyzed within the same real-time PCR. This dual detection allows normalizing the abundance of one qTag (representing chromosomal DNA from one specific mutant) to the total number of all qTags (which corresponds to chromosomal DNA from all strains) present in the same reaction. This internal control eliminates inaccuracies that can otherwise result from technical errors including incorrect DNA measurements or inaccurate pipetting. Third, all qTags are of similar length and of similar GC content and can be amplified with primers that hybridize upstream and downstream of each qTag (Figure 1.B and Figure S1). This permits quantification of their relative abundance even in samples that contain few bacteria (e.g. infected tissues, Figure 9.C). Fourth, none of the qTag sequences occur in the genomes of mycobacteria or animals, which prevents cross-hybridization of the TaqMan probes used for their detection. Fifth, the qTags have been incorporated into a vector system that permits rapid and efficient cloning of different expression plasmids by site-specific recombination (Figure 1.A).

To test the performance of the qTags we constructed three knockdown mutants and two reference strains and characterized their growth in three different media, their survival during oxidative stress, acid stress and starvation and their growth and survival in mice. We selected *icl1/2*, *rv3671c* and *prcBA* as targets for mutant construction because the depletion of their products cause well characterized phenotypes *in vitro* and during infection: inactivation of both ICLs prevented growth with a fatty acid as the primary carbon source [40], inactivation of Rv3671c decreased resistance to acid [41], and inactivation of PrcBA caused a slight growth impediment in 7H9, increased resistance to H_2_O_2_ [43] and decreased survival during starvation in PBS [27]. Previous reports also demonstrated that inactivation of ICL, Rv3671c and PrcBA respectively prevent growth, impair growth and persistence, or primarily decrease persistence of *Mtb* in mice [40]–[41], [43]. Our analyses of multi-strain cultures and multi-strain infections confirmed all of these phenotypes (Figures 3, 6, 8). They furthermore predicted (i) that the growth defect that results from silencing *prcBA* was amplified in Sauton's medium containing butyrate as the primary carbon source, (ii) that silencing of *rv3671c* caused a growth defect in Sauton's medium irrespectively of the carbon source, (iii) that one of the two reference strains we used (*Mtb* Erdman) was more resistant to H_2_O_2_ than the other (*Mtb* H37Rv), and (iv) that inactivation of ICL1 and ICL2 cleared *Mtb* from chronically infected mice. All of these predictions were confirmed in single-strain experiments (Figures 4, 7, and 10). These experiments, thus, demonstrated that real-time PCR based quantification of qTag-labeled mutants provides a sensitive and accurate approach to analyze several mutants simultaneously under a variety of conditions.

We also compared two approaches to measure the relative abundance of mutants in mouse lungs. In the first, bacterial chromosomal DNA was prepared after bacteria were recovered from lungs on agar plates (Figure 8). The advantage of this procedure is its high sensitivity, which allowed detection of mutants present at approximately 10^2^ CFUs per lung at time points when the reference strains achieved approximately 10^6^ CFUs per lung. The advantages of the second procedure, which uses DNA from total lung extracts (Figure 9), are that it allows to directly test if mutant titers change in the infected tissue and that it can provide information within a day or two after an animal has been sacrificed. This is significant because recovery of *Mtb* from lung extracts requires weeks of incubation. Its main disadvantage is the lower sensitivity compared to isolating DNA from bacteria grown on agar plates.

The primary goal of this study was to develop and validate a method to analyze several knockdown mutants simultaneously and we therefore did not attempt to improve complementation of *icl*-TetON or *rv3671c*-TetON. However, even though complementation of *icl*-TetON *in vitro* and *in vivo* was incomplete, the analyses of this mutant in single-strain and multi-strain mouse infections demonstrated that ICL activity is not only essential for *Mtb* to establish an infection in mice but also to persist during chronic infections (Figure 10). When transcription of *icl1* was repressed by removal of doxy, *icl*-TetON was cleared irrespectively of the time point at which doxy was removed. In contrast, *icl*-TetON persisted during single-strain infections in mice that received doxy throughout the infection.

While we have used the qTags to analyze conditional knockdown mutants they are just as suitable to analyze transposon mutants, deletion mutants, or point mutants and their complemented derivatives as well as different clinical isolates in multi-strain cultures or multi-strain infections. Furthermore, to our knowledge the qTag sequences do not occur in any other bacteria, which should facilitate their use in pathogens other than *Mtb*.

## Materials and Methods

### Plasmids, bacterial strains, media, reagents, and molecular biology techniques

The bacterial strains and plasmids used in this study are listed in Table S1. Cut and paste clonings were performed using standard procedures; BP and LR recombinase mixtures were purchased from Invitrogen and used according to the manufactures instructions. Unless otherwise noted, mycobacteria were grown in Middlebrook 7H9 liquid medium (Difco) containing 0.2% glycerol, 0.05% Tween80, 0.5% BSA, 0.2% dextrose, and 0.085% sodium chloride. To prepare 7H9 agar plates, we added 15 g/l bacto agar (Difco) to Middlebrook 7H9 medium with 0.5% glycerol supplemented with 10% oleic acid, albumin, dextrose, catalase (OADC; Middlebrook). Anhydrotetracyline (atc) was purchased from Sigma and 200 ng/ml were added to liquid media and agar plates where indicated.

### *In vitro* growth and stress exposure of multi-strain *Mtb* cultures

Bacteria were first grown in Middlebrook 7H9 with and without atc. At an optical density at 580 nm (OD_580_) of 0.8–1.2 bacteria were harvested by centrifugation, washed twice with PBS containing 0.05% Tween80, and centrifuged at 123×g to obtain single-cell suspensions whose optical density was adjusted to ∼1.0. Equal volumes of the single-strain single-cell suspensions were mixed to prepare 3 input cultures with atc and 3 input cultures without atc. To monitor growth in Middlebrook 7H9 and Sauton's medium (with 0.2% glycerol) or Sauton's medium modified to contain 0.1% butyrate as carbon source, 30 ml of the different media were inoculated with aliquots of the different input mixes at an OD_580_ of 0.01. The remainder of the input cultures was used to prepare genomic DNA [56].

To monitor survival during starvation, we inoculated the multi-strain input cultures at an OD_580_ of 0.05 in 5 ml PBS containing 0.05% Tween80 with and without atc. Aliquots of the PBS/Tween80 cultures were plated at different time points on Middlebrook 7H9 agar plates enriched with 10% OADC and containing atc. After incubation at 37°C for 4–5 weeks, we washed mycobacterial colonies off the agar plates, pelleted the bacteria by centrifugation, and isolated DNA.

For oxidative stress experiments we diluted the multi-strain input cultures in enriched 7H9 liquid medium to an OD_580_ of 0.05 and added 9 mM H_2_O_2_ or left the cultures untreated. Before H_2_O_2_ was added to the treatment cultures and after 4 h of H_2_O_2_ exposure, aliquots were spread on enriched 7H9 agar plates containing atc, and bacteria that had grown after incubation at 37°C were used for genomic DNA isolation. For low pH exposure, we diluted input mixes in enriched 7H9 liquid medium of pH 4.5 containing 0.02% tyloxapol to an OD_580_ of 0.05. Atc was added to cultures that had been pre-grown with atc. Directly after dilution and after 3 days of incubation at 37°C, we spread culture aliquots on enriched 7H9 agar plates containing atc to obtain bacterial colonies for genomic DNA isolation.

### Real-time PCR

We diluted genomic mycobacterial DNA in 10 mM Tris/HCL, pH 8.0 and performed quantitative real-time PCR analyses using a LightCycler480 II (Roche). The probes and primers used are shown in Figure S1 and Table S2. The PCR for the variable region of an individual DNA tag and the common region were performed in the same reaction mix detecting the fluorescence signals of the FAM and LC670 labeled probes respectively. We determined the crossing points of the amplification curves for the variable and the common DNA tag region for each sample by calculating the second derivative maximum (LCS480 1.5.0.39 software). Serial dilutions of defined copy numbers of individual qTags were included for each real-time PCR to generate standard curves, which were used to calculate the absolute copy numbers of the variable and common region in each PCR reaction mix. Next, we calculated, what percentage of the total DNA tag copies can be attributed to a specific DNA tag in the sample in the same PCR reaction mix. These values were then normalized to the *Mtb* H37Rv reference strain and plotted as “relative abundance”.

### Mouse infections

All mouse procedures performed in this study were conducted following the National Institutes of Health guidelines for housing and care of laboratory animals and performed in accordance with institutional regulations after protocol review and approval by the Institutional Animal Care and Use Committee of Weill Cornell Medical College (protocol #0802-713A). Eight to ten weeks old female C57BL/6 mice were infected with *Mtb* using aerosols produced by nebulizing 5 ml of a bacterial single-cell suspension in PBS at ∼1.5×10^8^ bacilli/ml (single-strain infections) or ∼8×10^8^ bacilli/ml (multi-strain infections). Doxy was administered using chow containing 2000 ppm doxy (Research Diets). At the indicated time points, lungs and spleens were removed, homogenized in PBS, and plated on 7H9 agar containing atc. We determined bacterial loads of infected organs by counting colony-forming units. For multi-strain infections, we also plated lung homogenates to obtain bacterial colonies for the isolation of genomic DNA.

## Supporting Information

Figure S1 **Sequence of the qTags used in this study.** Sequences that bind or correspond to the primers and probes used for real-timer PCR are shown in red, blue, and brown.
(TIF)

Table S1 **Bacterial strains and plasmids.**
(DOC)

Table S2 **TaqMan probes and primers.**
(DOC)
