# Supplementary material for: Simultaneous Analysis of Multiple Mycobacterium tuberculosis Knockdown Mutants In Vitro and In Vivo
Source: PLoS One. 2010 Dec 22;5(12):e15667. doi: 10.1371/journal.pone.0015667 (PMC3008731; doi:10.1371/journal.pone.0015667)
Supplement: Table S1 — Bacterial strains and plasmids. (DOC) [file pone.0015667.s002.doc]

**Table S1**. Bacterial strains and plasmids

| E. coli | Description | Source or reference |
| --- | --- | --- |
| Mach1^TM^-T1^R^ | F– Φ80lacZΔM15 ΔlacX74 hsdR(rK–, mK+) ΔrecA1398 endA1 tonA*;* used as general cloning strain | Invitrogen |
| DB3.1^TM^ | F– gyrA462 endA1 Δ(sr1-recA) mcrB mrr hsdS20(rB–, mB–) supE44 ara-14 galK2 lacY1 proA2 rpsL20(SmR) xyl-5 λ– leu mtl1; used to clone and propagate *ccdB* containing plasmids | Invitrogen |
| Mtb | Description | Source or reference |
| H37Rv | wild type | Gift from R. North, Trudeau Institute |
| H37Rv-q26 | Kan^r^, hyg^r^; *Mtb* H37Rv transformed with pGMCKq26-10M-hyg; used as H37Rv reference in multi-strain experiments | This study |
| Erdman | wild type | Gift from J.D. McKinney, Ecole Polytechnique Federale de Lausanne |
| Erdman-q17 | Strep^r^; *Mtb* Erdman transformed with pGMCSq17-10M0X; used as Erdman reference in multi-strain experiments | This study |
| Δ*icl1* Δ*icl2* | Kan^r^, hyg^r^; *Mtb* Erdman derivative in which *icl1* and *icl2* have been deleted | Munoz-Elisa et al. (2005) Nat Med 11: 638-644 |
| *icl*-TetON | Kan^r^, hyg^r^, strep^r^; *Mtb* Δ*icl1* Δ*icl2* transformed with pGCMSq22-10M1-*icl1* | This study |
| Δ*rv3671c* | Hyg^r^; *Mtb* H37Rv derivative in which *rv3671c* has been deleted. | S. Ehrt, unpublished |
| *rv3671c*-TetON | Kan^r^, hyg^r^; *Mtb* Δ*rv3671c* transformed with pGMCKq20-10M1-*rv3671c*-SD2 | This study |
| P_myc1_tetO::*prcBA* | Hyg^r^; *Mtb* H37Rv derivative in which the native *prcBA* upstream region has been replaced | Gandotra et al (2007) Nat Med 13: 1515-1520 |
| *prcBA*-TetON | Kan^r^, hyg^r^; *Mtb* P_myc1_*tetO*:*prcBA* transformed with pGMCKq19-10M0X | This study |
|  |  |  |
| Plasmid | Description | Source or reference |
| pDO23A | Amp^r^; used to clone target genes by BP recombination | This study |
| pEN41A-T10M | Amp^r^; entry plasmid containing expression cassette for tetR-10 [1] | This study |
| pEN12A-P1 | Amp^r^; entry plasmid containing P_myc1_*tetO* [2] | This study |
| pDE43-MCKq19 | Cm^r^, kan^r^; destination plasmid containing qTag-19 | This study |
| pDE43-MCSq17 | Cm^r^, strep^r^; destination plasmid containing qTag-17 | This study |
| pDE43-MCKq20 | Cm^r^, kan^r^; destination plasmid containing qTag-20 | This study |
| pDE43-MCSq22 | Cm^r^, strep^r^; destination plasmid containing qTag-22 | This study |
| pDE43-MCKq26 | Cm^r^, kan^r^; destination plasmid containing qTag-26 | This study |
| pGMCKq26-10M-hyg | Kan^r^, hyg^r^; TetR-10 expression plasmid containing qTag-26; integrates into attachment site of mycobacteriophage L5 | This study |
| pGMCSq17-10M0X | Strep^r^; TetR-10 expression plasmid containing qTag-17; integrates into attachment site of mycobacteriophage L5 | This study |
| pGMCKq20-10M1-*rv3671c*-SD2 | Kan^r^; regulated expression plasmid for Rv3671c containing qTag-20; integrates into attachment site of mycobacteriophage L5 | This study |
| pGMCKq19-10M0X | Kan^r^; TetR-10 expression plasmid containing qTag-19; integrates into attachment site of mycobacteriophage L5 | This study |
| pGMCSq22-10M1-*icl1* | Strep^r^; regulated expression plasmid for ICL1 containing qTag-22; integrates into attachment site of mycobacteriophage L5 | This study |
