# Supplementary material for: Simultaneous Analysis of Multiple Mycobacterium tuberculosis Knockdown Mutants In Vitro and In Vivo
Source: PLoS One. 2010 Dec 22;5(12):e15667. doi: 10.1371/journal.pone.0015667 (PMC3008731; doi:10.1371/journal.pone.0015667)
Supplement: Table S2 — TaqMan probes and primers. (DOC) [file pone.0015667.s003.doc]

**Table S2**. TaqMan probes and primers

| Amplicon | Forward primer | Reverse primer | Probe |
| --- | --- | --- | --- |
| common | GGCCTAGCTGGCATCGGTA | ACACCAGATGGACACGCTCC | LC670- TGCCGCGCAACCATGTAGTAGTCC-BBQ |
| variable-17 | GAAGTCCAACACGCCGCT | ACCGTTGCGCAGGAATTC | 6FAM-CCATCCGCACCACCCTAGTTCGAG-BHQ1 |
| variable-19 | GGCTAGATGTGCAACGCATG | GCGTCAGTGAAATAGCGCTCT | 6FAM-AGTCGCCAATCCACGGCCGA-BHQ1 |
| variable-20 | AACTTCTTCAAGAGGCTGCCG | ATCAGCAGCAAGAACGCCAT | 6FAM-CCCGCGCCGATACCATGCT-BHQ1 |
| variable-22 | GCCGAGCAGTGCACGTACT | CCTTCAGGCGTTGATGTCG | 6FAM-CGTGTGACCTGAGCCGCGAATACA-BHQ1 |
| variable-26 | CTTCTACAACAACCCGCTGCT | ACTACGCCATGTCCGTCGTC | 6FAM-TCGTTACGCCGCCTCCTTGGAT-BHQ1 |
